# Epstein-Barr virus is not detected in mucosal lichen planus

**DOI:** 10.4317/medoral.22617

**Published:** 2018-09-28

**Authors:** Karin Danielsson, Elisabet Nylander, Mats Sjöström, Majid Ebrahimi

**Affiliations:** 1 Department of Odontology, Umeå University, Umeå, Sweden; 2 Dept. of Public health and Clinical Medicine, Dermatology and Venereology, Umeå University, Umeå, Sweden

## Abstract

**Background:**

Lichen planus (LP) is a chronic inflammatory, immunological, mucocutaneous disease can affect skin, genital and oral mucosa. Oral lichen planus (OLP) is the most common noninfectious, chronic inflammatory oral disease affecting 1-2% of the general adult population. World Health Organization (WHO) classifies OLP as a potentially malignant disorder. Epstein Barr virus or human herpesvirus-4, is a member of the herpes virus family and one of the most ubiquitous viruses known to human, infecting approximately 90% of the world’s adult population. The virus often infects B lymphocytes resulting in a wide spectrum of mucocutaneous and systemic diseases, ranging from mild lesions to aggressive malignancies. The aim of this study was to investigate expression of the EBV encoded RNAs EBER1 and EBER2 in oral and genital lichen planus and compare results with normal tissues in situ hybridization which is considered the golden standard for detection of EBER.

**Material and Methods:**

A total of 68 biopsies, 25 oral LP, 26 genital LP, 10 oral controls and finally 7 genital controls were analysed using situ hybridization.

**Results:**

All samples had RNA as shown by the control slide, whereas no case contained neither EBER1 nor EBER2.

**Conclusions:**

Based on results from our study EBV is not involved in aetiology of lichen planus.

** Key words:**Mucosal lichen planus, Epstein - Barr virus.

## Introduction

Lichen planus (LP) is a chronic inflammatory, immunological, mucocutaneous disease with different clinical appearances (1,2). LP can affect skin, genital and oral mucosa, scalp and nails (3). Oral lichen planus (OLP) is the most common noninfectious, chronic inflammatory disease affecting 1-2% of the general adult population (4) but the range of prevalence varies among different populations in the world (3). The disease is more common among women than men and most often occurs in middle-aged patients (3)The oral mucosa can be the only site of involvement (1). However, in a recently published paper it has been shown that the vast majority of patients have multifocal lesions (5). LP has been characterised as a T-cell mediated and autoimmune disease, whereas the exact trigger mechanisms of LP development are not yet fully understood (6). As with LP, the aetiology of OLP is not yet fully understood (3), but autoimmunity could be a causative or contributing factor to OLP (7,8). It has been shown that LP may be associated with other autoimmune diseases, such as ulcerative colitis, vitiligo and myasthenia gravis (4). OLP lesions can affect any mucosal surface in oral cavity but the most common affected locations are the buccal mucosa. The clinical expression varies between six different variants: papular, plaque-like, bullous, atrophic, reticular and erosive (2). Histologically OLP is characterized by a band like massive infiltrate of lymphocytes in the superficial lamina propia. It is also characterized by hyperkeratosis, degeneration of basal cell layer and possibly saw tooth appearance or absence of rete ridges (2). In contrast to dermal LP, mucosal LP is considered to follow a more chronic direction and therefore has only a small chance for spontaneous regression (9).

World Health Organization (WHO) classifies OLP as a potentially malignant disorder (2). The malignant potential of OLP, however, is highly controversial (9,10). Former studies have shown that OLP can develop into squamous cell carcinoma (SCC) but the exact carcinogenetic mechanism is still not discovered.

EBV or human herpesvirus-4, is a member of herpes virus family and one of the most ubiquitous viruses known to human, infecting approximately 90% of the world’s adult population. Like all herpes viruses EBV may remain in the host organism for life, but in most cases does not cause any harm in healthy carriers (11). The virus often infects B lymphocytes resulting in a wide spectrum of mucocutaneous and systemic diseases, ranging from mild lesions to aggressive malignancies (12). The virus only affects humans and two types have been identified: Type A or 1 and type B or 2. Epstein-Barr virus can either be in a latent phase within memory B cells or in a lytic, active phase responsible for viral replication (12).

Primary EBV infection occurring in children is often asymptomatic (13). In contrast, primary EBV infection in older patients causes infectious mononucleosis (IM) in 30% to 50% of infected persons (14). In recent years possible viral infection such as Hepatitis C virus, Human Herpes virus-6 and EBV has been linked to aetiology of OLP (15).

Epstein Barr virus encoded RNA can be detected by different methods but Epstein Barr virus-encoded RNA in situ hybridization is considered the gold standard for detecting and localizing latent Epstein Barr virus in tissue samples (16,17). The aim of this study was to investigate expression of the EBV encoded RNAs EBER1 and EBER2 in oral and genital lichen planus and compare results with normal tissues using golden standard of in situ hybridization. 

## Material and Methods

A total of 68 biopsies were collected in this study. These biopsies consisted of 25 oral LP, 26 genital LP, 10 oral controls (OC) and finally 7 genital controls (GC) ( Table 1). All 51 samples of LP were in an active state of inflammation, showing a well-defined inflammatory infiltrate. None of the OLP biopsies had histologically dysplastic features.

**Table 1:**
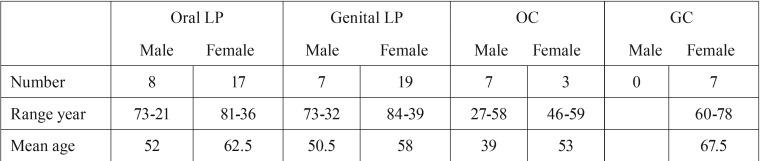
Data on patients and controls regarding gender and age.

Samples, were retrieved from the archives at the Department of Clinical Pathology, University Hospital, Umeå. The study was approved by the Ethical Review Board at Umeå University (dnr 2013-252-32M).

For EBER-in situ hybridisation (EBER-ISH) a commercially available kit was used. From each samples two sections were cut and in one of the sections EBER1 and EBER 2 was detected using the Inform EBER Probe (800-2842, Ventana Medical Systems, Roche Diagnostics GmbH, Mannheim Germany). At the same time section number 2 was incubated with an RNA Positive Control Probe (800-2846, Ventana) in order to make sure mRNA was present in the samples. The NBT-BCIP Detection Chemistries (800-092, Roche Diagnostics GmbH, Mannheim, Germany) was used for detection. Stainings were performed using a Bench Mark Ultra (Ventana Medical Systems, Inc, Tucson, AZ, USA) according to the supplier’s protocols.

## Results

All biopsies were assessed by to investigators (ME and KD) independently. In cases of disagreement slides were discussed in a joint session until consensuses was reached.

**Figure 1:**
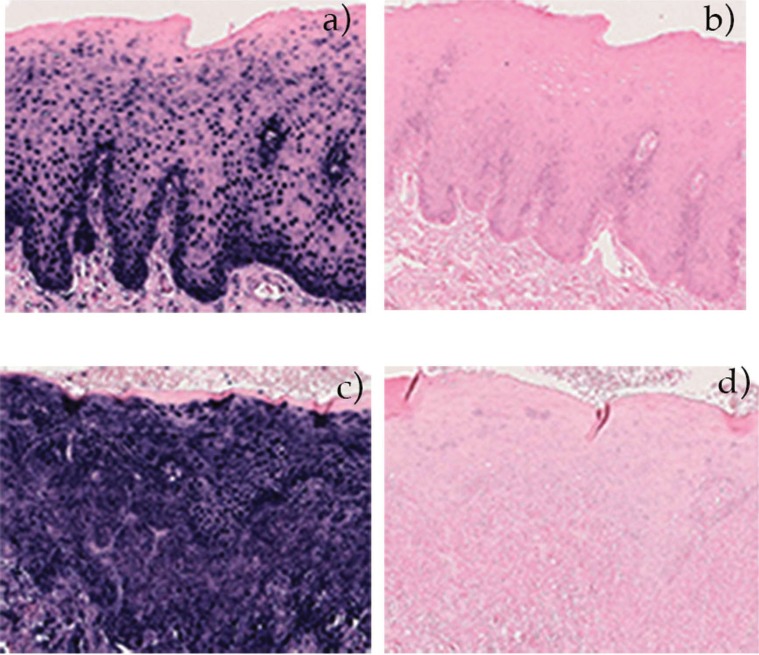
Representative slides from OLP and control. Slides a) OLP and C) control show occurrence of RNA whereas b) OLP and d) control contained neither EBER1 nor EBER2.

All samples had RNA as shown by the control slide, whereas no case contained neither EBER1 nor EBER2 (Fig. 1).

## Discussion

Since the discovery of the EBV, the first identified human tumour virus by Epstein (Epstein *et al.*, 1964) this virus has been the subject of study in many kinds of cancer. The virus often infects B lymphocytes resulting in a wide spectrum of mucocutaneous and systemic diseases, ranging from mild lesions to aggressive malignancies (12).

EBV is one of the most ubiquitous viruses known to human infecting approximately 90% of the world’s adult population. Like all herpes viruses EBV may remain in the host organism for life, but in most cases it does not cause any harm in healthy carriers (11). The oral cavity is a primary site for transmission and persistence of EBV. EBV can also be detected in other anatomical sites, and is found in blood circulating B cells in healthy carriers (18). In order to study presence of EBV one must target the specific tissue or organ with specific method.

EBER represent two RNA species, EBER1 and EBER2, encoded from two separate but homologous viral genes. EBER transcripts are expressed in latently infected cells at levels approaching a million copies per cell (19). Because EBER transcripts are naturally amplified, they represent a reliable target for detecting and localizing EBV in tissue sections by in situ hybridization (20). This may explain why our results differ from the previous study.

Reviewing literatures reveals rather few papers with divergent results depending on different method and choice of tissue. In this study in contrary to all other studies we could not detect occurrence of EBER expression in epithelium in oral and genital lichen planus as well as healthy controls. Yildirim and co-workers used immunohistochemistry, staining OLP slides with EBV primary antibody EBV/LMP-Ab-1 and found that 35% of OLP patients were positive and suggested that EBV may play a role in the ethiopathogenesis of OLP 

By using PCR or ELISA some investigators detect antibodies against EBV in approximately 25% of LP patients (21-23) while others have found as high as 62% EBV positive tissue, 18% in saliva, 17% in exfoliated cells and 8% of plasma , using nested PCR (24). Still, no correlation between OLP and EBV was seen.

Using different PCR techniques, requires initial homogenizing of tissue and extraction of DNA. This eliminates the possibility of determining which cell type harboured the virus, and allows for false positive results due to contamination with laboratory EBV DNA or EBV-infected lymphocytes in the specimen. The positive result for EBV by solution PCR could have been due to EBV-infected lymphocytes rather than LP epithelium.

Despite intense research in the field of lichen planus there are some crucial questions that remain unanswered among those premalignant potential of lichen planus and some authors investigate expression of EBV in lichen planus and somehow connect it to LP´s pre- malignant potential.

Results of our study is based on Epstein Barr virus-encoded RNA in situ hybridization which is considered the golden standard for detecting and localizing latent Epstein Barr virus in tissue samples (16,17).

EBER transcripts are systematically expressed in every EBV-infected tumour, and in lymphoid tissues taken from patients with infectious mononucleosis, and in the rare infected cell representing normal flora in healthy virus carriers. In fact, oral hairy leukoplakia is the only EBV-related lesion that lacks EBER (25).

Based on results from our study EBV is not involved in aetiology of lichen planus.
